# CD44 Antibodies and Immune Thrombocytopenia in the Amelioration of Murine Inflammatory Arthritis

**DOI:** 10.1371/journal.pone.0065805

**Published:** 2013-06-13

**Authors:** Patrick J. Mott, Alan H. Lazarus

**Affiliations:** 1 The Canadian Blood Services, Toronto, Ontario, Canada; 2 Keenan Research Centre in the Li Ka Shing Knowledge Institute of St. Michael’s Hospital, Toronto, Ontario, Canada; 3 Departments of Medicine University of Toronto, Toronto, Ontario, Canada; 4 Laboratory Medicine & Pathobiology, University of Toronto, Toronto, Ontario, Canada; University Hospital Jena, Germany

## Abstract

Antibodies to CD44 have been used to successfully ameliorate murine models of autoimmune disease. The most often studied disease model has been murine inflammatory arthritis, where a clear mechanism for the efficacy of CD44 antibodies has not been established. We have recently shown in a murine passive-model of the autoimmune disease immune thrombocytopenia (ITP) that some CD44 antibodies themselves can induce thrombocytopenia in mice, and the CD44 antibody causing the most severe thrombocytopenia (IM7), also is known to be highly effective in ameliorating murine models of arthritis. Recent work in the K/BxN serum-induced model of arthritis demonstrated that antibody-induced thrombocytopenia reduced arthritis, causing us to question whether CD44 antibodies might primarily ameliorate arthritis through their thrombocytopenic effect. We evaluated IM7, IRAWB14.4, 5035-41.1D, KM201, KM114, and KM81, and found that while all could induce thrombocytopenia, the degree of protection against serum-induced arthritis was not closely related to the length or severity of the thrombocytopenia. CD44 antibody treatment was also able to reverse established inflammation, while thrombocytopenia induced by an anti-platelet antibody targeting the GPIIbIIIa platelet antigen, could not mediate this effect. While CD44 antibody-induced thrombocytopenia may contribute to some of its therapeutic effect against the initiation of arthritis, for established disease there are likely other mechanisms contributing to its efficacy. Humans are not known to express CD44 on platelets, and are therefore unlikely to develop thrombocytopenia after CD44 antibody treatment. An understanding of the relationship between arthritis, thrombocytopenia, and CD44 antibody treatment remains critical for continued development of CD44 antibody therapeutics.

## Introduction

Rheumatoid Arthritis (RA) is a common autoimmune disorder that results in inflammation of the synovial joints of patients. Though RA affects approximately 1% of the population, and is classified as an autoimmune disorder, the molecular event(s) which initiate the evasion of tolerance remain speculative and unconfirmed [1], [2]. However, after tolerance has been evaded, lymphocytes are thought to be recruited to the joint [1], [2], [3], where they are activated and form follicle-like structures similar to germinal centres found in the peripheral lymphoid tissues [4]. Local activation of the recruited leukocytes and lymphocytes results in the release of proinflammatory mediators such as TNF-α, IL-1, and IL-6 [5], as well as the production of autoantibodies (such as anti-cyclic citrullinated peptide antibodies and rheumatoid factors) [6], [7]. Autoantibodies can activate the complement cascade [8], and promote the recruitment of leukocytes and further drive local inflammation of the synovial tissue. In addition to lymphocytes, other cells including mast cells [9], macrophages [10], and fibroblast-like synoviocytes (FLS) [11] all appear to play important roles in the progression of RA. Given the inability to prevent the initiation of RA in patients, much research has focused on developing treatments to prevent or alleviate inflammation of the joints. In several different mouse models of inflammatory arthritis, anti-CD44 antibodies have been shown to have potent anti-inflammatory effects [12], [13], [14], though their exact mechanism remains unclear.

CD44 is a transmembrane protein expressed on almost all nucleated cells in mice and humans [15] and can function as a cellular receptor for hyaluronan (HA) [16]. With respect to HA, CD44 is thought to exist in one of three different conformations: HA non-binding, inducible HA binding, and constitutive HA binding [17]. In this paradigm, antibodies to CD44 are thought to block lymphocyte [18] or neutrophil [19] binding to HA presented at the site(s) of inflammation [20]. Some antibodies directly block recognition of HA by CD44 (the KM-group of antibodies used here) [21], while others do not block CD44-HA binding. Recent work has noted that some CD44 antibodies are capable of depleting granulocytes [14] and platelets [14], [22] from circulation, raising the possibility that CD44 antibody treatment may have effects through depletion of cells expressing CD44, such as lymphocytes, granulocytes, or other target population(s).

A target population of interest are platelets, which are now thought to contribute to the inflammatory environment of the rheumatoid synovium [23]. The involvement of platelets in RA was first supported by the observation of platelets in the rheumatic synovial fluid (SF) [24], which when healthy is considered to be unoccupied by cells or cellular fragments. More recent work by Boilard *et al.* showed that platelet microparticles (MPs) were more frequently found in the SF in RA patients compared to osteoarthritic patients [25]. These MPs were speculated to be formed when platelets are exposed to collagen, and were capable of activating FLS to produce IL-6 and IL-8. In the K/BxN model of inflammatory arthritis, depletion of platelets (thrombocytopenia) with a polyclonal antibody preparation targeting platelet glycoprotein Ib (GPIb) in arthritic mice resulted in a significant reduction of inflammation and histopathologic scores relative to arthritic mice. Platelets have also been shown to enhance vascular permeability in the context of inflammatory arthritis [26], an effect in stark contrast to their best known role of maintaining hemostasis. Depletion of platelets with the same anti-GPIb preparation reduced the number of circulating platelets, as well as reducing vascular leakage in the arthritic joints [26]. Platelets have also been shown to promote inflammation through a pathway where platelet produced prostaglandin H2 (PGH_2_), stimulated FLS to produce prostacyclin, promoting inflammation in the context of arthritis [27].

Antibody-induced thrombocytopenia can be caused by a number of different monoclonal anti-platelet antibodies [28], and we have recently observed that CD44 antibodies can also cause thrombocytopenia in mice [22]. In this report we therefore questioned whether the therapeutic effect of CD44 antibodies in inflammatory arthritis might be primarily due to their thrombocytopenic effect. We treated mice with a panel of anti-CD44 antibodies in comparison to anti-platelet antibody, and found that while the CD44 antibodies induced thrombocytopenia and were effective in ameliorating arthritis at the concentrations used, the therapeutic effect did not appear to be closely related to the severity or length of the thrombocytopenia. Critically, CD44 antibody treatment was able to reverse established inflammatory arthritis while anti-platelet antibody was without effect.

## Materials and Methods

### Mice and K/BxN Sera

KRN TCR transgenic mice on the C57BL/6 background were originally obtained from Drs. Diane Mathis and Christophe Benoist from the Institut de Genetique et de Biologie Moleculaire et Cellulaire (IGBMC) CNRS INSERM France [29]. C57BL/6 mice were obtained from Charles River (St-Constant, QC). Male NOD/ShiLtJ mice were obtained from Jackson Laboratory (Bar Harbor, ME), and were bred with female heterozygous KRN +/− females to maintain a breeding pool as previously described [30]. To produce K/BxN serum, strongly arthritic K/BxN mice (clinical score greater than 9) were anesthetized and bled by cardiac puncture as a terminal procedure. Individual serum samples from at least 25 mice were frozen and stored at −80°C until they were combined into a pooled lot of K/BxN serum. The efficacy of each lot of pooled K/BxN serum was assessed through independent dose response experiments.

### Ethics Statement

All animal work was conducted in the St. Michael’s Hospital vivarium, and approved by the St Michael’s Hospital Animal Care and Committee. All care was taken to prevent or minimize any pain or suffering.

### Antibodies

CD44 antibodies were obtained as follows: IM7.8.1 (IM7; BD Biosciences, Cat No. 553131 and BioXcell, Cat No. BE0039), KM201 (Fitzgerald Industries International, Cat No. 10R-CD44gMS), KM114 (BD Biosciences, Cat No. 558739), KM81 (Cedarlane Labs, Cat No. CL8944AP), and 5035-41.1D (Lifespan Biosciences, Cat No. 558739). IRAWB14.4 was obtained as a kind gift from Dr Katalin Mikecz (Rush Medical Centre, Chicago). Normal rat IgG was obtained from Caltag Laboratories (Burlingame, CA. Cat No. 10700). Rat anti-mouse platelet antibody (MWReg30) was obtained from BD Biosciences (Mississauga, ON. Cat No. 553847).

### Induction of Arthritis, Clinical Score, and Platelet Counts

Pooled K/BxN serum sufficient to cause severe arthritis (approximate clinical score of 8 or more) in 100% of recipient mice was injected into the tail vein of C57BL/6 mice (Day 0). Clinical score, ankle measurements and platelet counts were performed daily commencing just prior to K/BxN serum injection. Platelet counts were determined using a Z2 Particle Counter (Beckman Coulter, Mississauga, ON) as previously described by us [31]. Ankle widths (in millimetres) of the hind paws were measured at the widest point (the malleoli) with the legs fully extended, using a digital caliper. Clinical scoring of inflammation and swelling was also recorded on a scale of 0–3 (per paw, reported as the sum of all four paws). A clinical score of 1 indicated mild swelling that did not noticeably change the shape of the paw, 2 indicated moderate swelling which modified the foot and joint to be of approximately equal width through-out, and 3 indicated severe swelling which reversed the normal “v-shape” of the foot when examined from below the foot [30].

### Arthritis Treatment

Anti-CD44 and anti-platelet antibodies were diluted in PBS and injected (200 uL) intraperitoneally (IP) two hours prior to arthritis induction. Anti-platelet antibody was administered at a dose of 2 ug per injection, while CD44 antibodies (or control rat IgG) were administered at doses of 150 ug/injection based upon the work of several investigators [12], [13], [14]. In experiments where anti-CD44 or anti-platelet antibody was given on more than one day, the additional injections were administered after the daily bleed for platelet counts and arthritis assessment.

To examine a sustained depletion of blood platelets, mice were injected with 2ug of anti-platelet antibody daily, commencing two hours before the induction of arthritis, for a total of ten days.

To induce an increase in platelet counts (rebound thrombocytosis), mice were treated with either a single dose of IM7 (150ug, day 0) or three daily doses of anti-platelet antibody (3 days×2ug, on days 0, 1, and 2) and the platelet count monitored daily. K/BxN serum was injected on day 5.

To examine the ability of anti-CD44 vs. anti-platelet antibody to reverse ongoing disease, arthritis was induced on Day 0 and IM7 (150ug, IP) or anti-platelet antibody (2ug, IP) injected on day 5. To ensure a level of thrombocytopenia similar to that induced by IM7, a second and third injection of anti-platelet antibody was administered on days 5 and 6. Mice were then monitored every 24 hours until day 10, to ensure platelet counts returned to pre-treatment levels.

### Statistical Analyses

**Figure 1:**
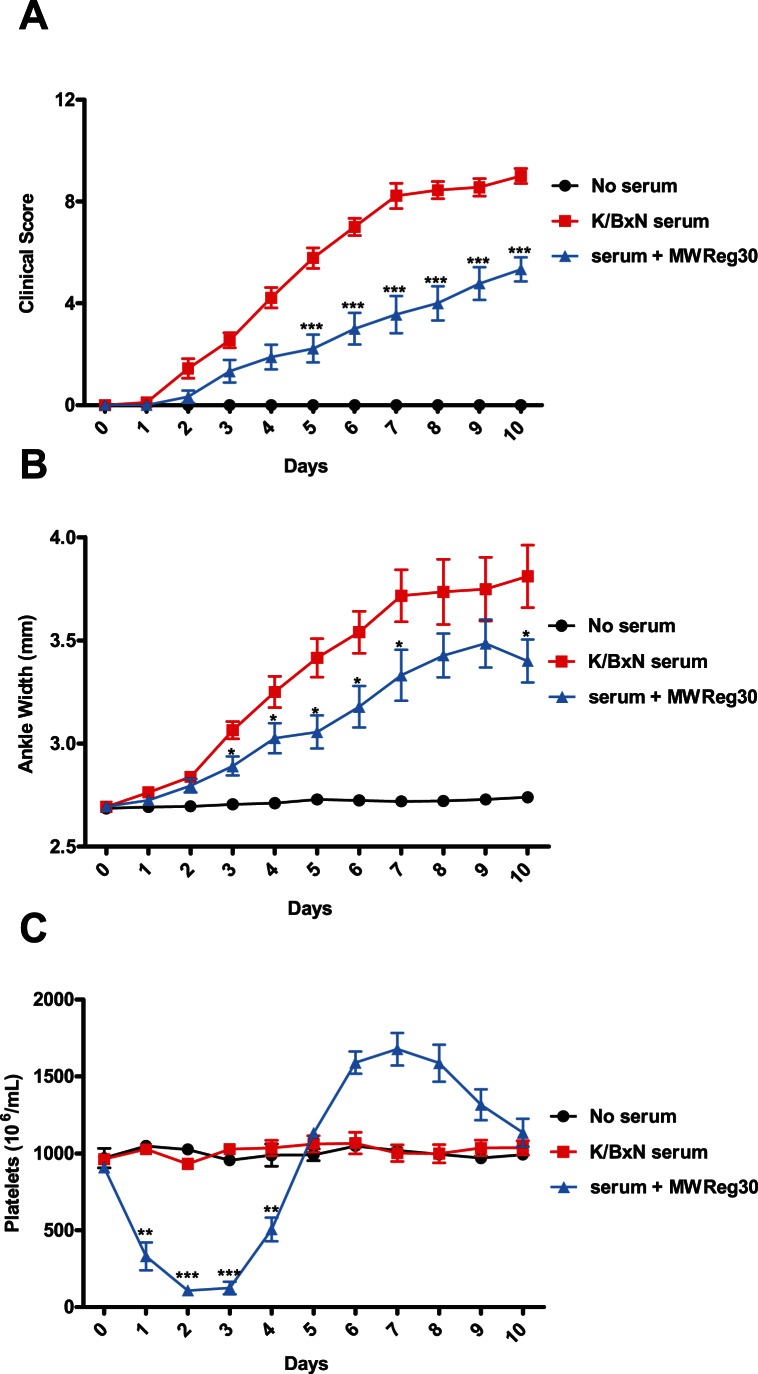
MWReg30, a platelet-depleting antibody, partially protects against serum-induced arthritis. C57BL/6 mice were assessed for platelet and arthritis measurements on day 0. The group receiving MWReg30 (blue) was injected with 2ug on days 0, 1, and 2 (every 24 hours). Arthritis was induced by injecting mice with 160uL of K/BxN serum. Clinical scoring and platelet counts were taken every 24 hours for 10 days. Platelet depletion reduced the severity of arthritis relative to K/BxN controls (red), as measured by clinical scoring (A) and ankle width measurements (B). C) MWReg30 induced significant thrombocytopenia. (n = 9 mice from 3 separate experiments) *P<.05, **P<0.01, ***P<0.001 by Student’s T-test.

**Figure 2:**
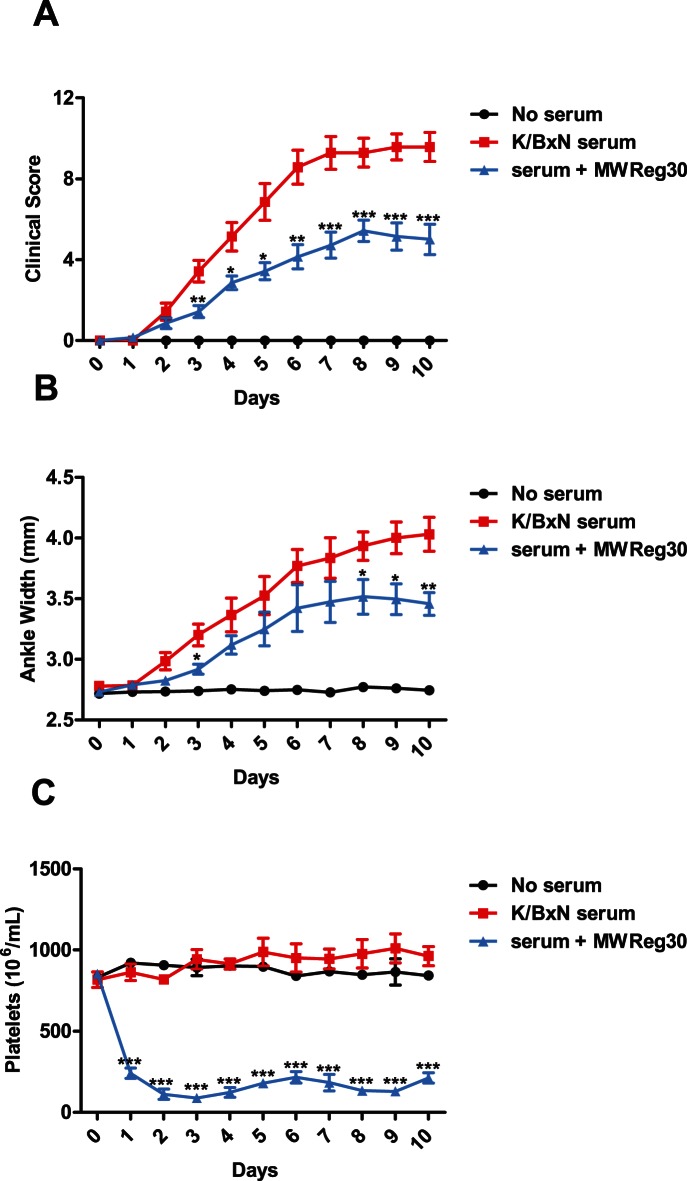
Continuous treatment with MWReg30 does not increase the protection against development of serum-induced arthritis. C57BL/6 mice were assessed for baseline measurements on day 0. MWReg30-treated mice were then injection with 2ug of MWReg30 (blue), and arthritis induced normally 2 hours later. Clinical scoring and platelet counts were taken every 24 hours for 10 days, and mice were treated with 2ug of MWReg30 every 24 hours (following arthritis and platelet measurements). Platelet depletion reduced the severity of arthritis relative to K/BxN controls (red squares), as measured by Clinical Scoring (A) and ankle width measurements (B). C) MWReg30 induced significant thrombocytopenia throughout the entire experiment. (n = 7 mice from 3 separate experiments) *P<.05, **P<0.01, ***P<0.001 by Student’s T-test.

**Figure 3:**
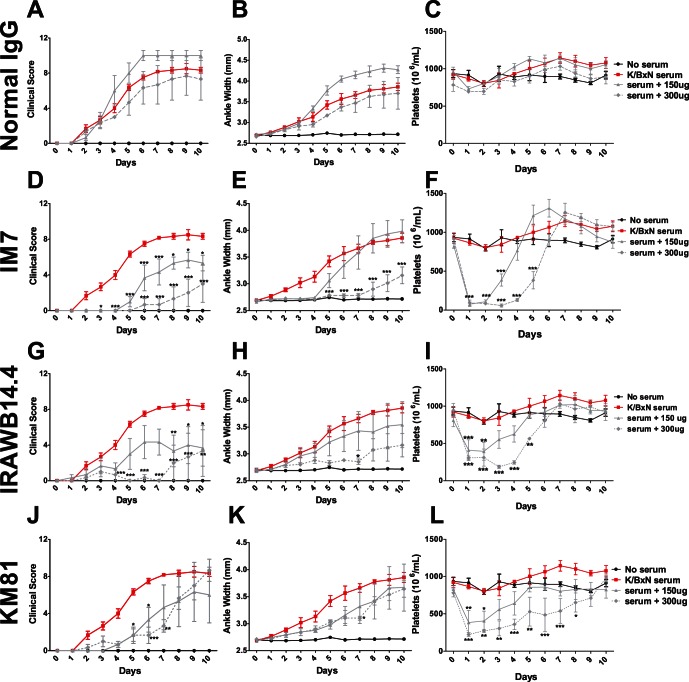
Anti-CD44 antibodies which cause severe thrombocytopenia have a significant therapeutic effect on inflammatory arthritis. C57BL/6 mice were assessed for baseline measurements on Day 0. Mice were pre-treated with a 150 µg dose (solid grey lines) of the indicated antibody on Day 0, then injected with K/BxN serum. Mice receiving only K/BxN serum (red lines) were also injected at this time. Mice receiving a second 150 µg dose (for a total of 300 µg) were injected with this second dose on Day 2 (dotted grey lines). Arthritis and platelet counts were performed every 24 hours for 10 days. Normal Rat IgG controls did not significantly reduce inflammation (A,B) or platelet (C) counts. IM7 (D,E) ameliorated arthritis, and induced a severe thrombocytopenia (F), with an additive effect observed in the group receiving a total dosage of 300 µg. IRAWB14.4 ameliorated arthritis (G,H), and induced a severe thrombocytopenia (I), with a slight additive effect observed in the group receiving a total dosage of 300 µg. KM81 delayed the onset of inflammation (J,K), and induced a severe thrombocytopenia (L), though no significant additive effect was observed in the group receiving a total dosage of 300 µg. (For groups receiving 150 µg or 300 µg of anti-CD44 treatment, n = 3 mice from 3 experiments. For the group receiving only K/BxN serum, n = 6 mice from 3 experiments) *P<.05, **P<0.01, ***P<0.001 by two-way ANOVA.

**Figure 4:**
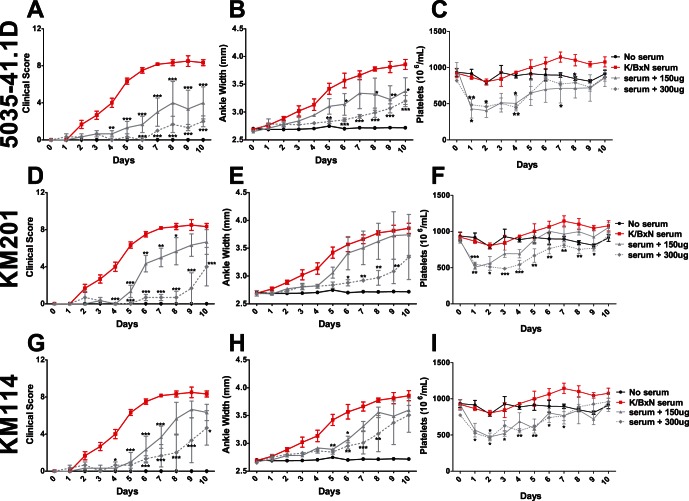
Anti-CD44 antibodies which cause intermediate thrombocytopenia have a significant therapeutic effect on inflammatory arthritis. C57BL/6 mice were assessed for baseline measurements on Day 0. Mice were pre-treated with a 150 µg dose (solid grey lines) of the indicated antibody on Day 0, then injected with K/BxN serum. Mice receiving only K/BxN serum (red lines) were also injected at this time. Mice receiving a second 150 µg dose (for a total of 300 µg) were injected with this second dose on Day 2 (dotted grey lines). Arthritis and platelet counts were performed every 24 hours for 10 days. 5035-41.1D was very effective in ameliorating inflammation (A,B), but only induced a moderate thrombocytopenia (C), a slight additive effect was observed in the group receiving a total of 300 µg. KM201 effectively ameliorated inflammation (D,E), with a moderate thrombocytopenia (F), and a significant additive effect was observed in the group receiving a total of 300 µg. KM114 was partially effective in ameliorating inflammation (G,H), with an intermediate thrombocytopenia (I), and no significant additive effect in the group receiving a total of 300 µg. (For groups receiving 150 µg or 300 µg of anti-CD44 treatment, n = 3 mice from 3 experiments. For controls receiving only K/BxN serum, n = 6 mice from 3 experiments) *P<.05, **P<0.01, ***P<0.001 by two-way ANOVA.

**Figure 5:**
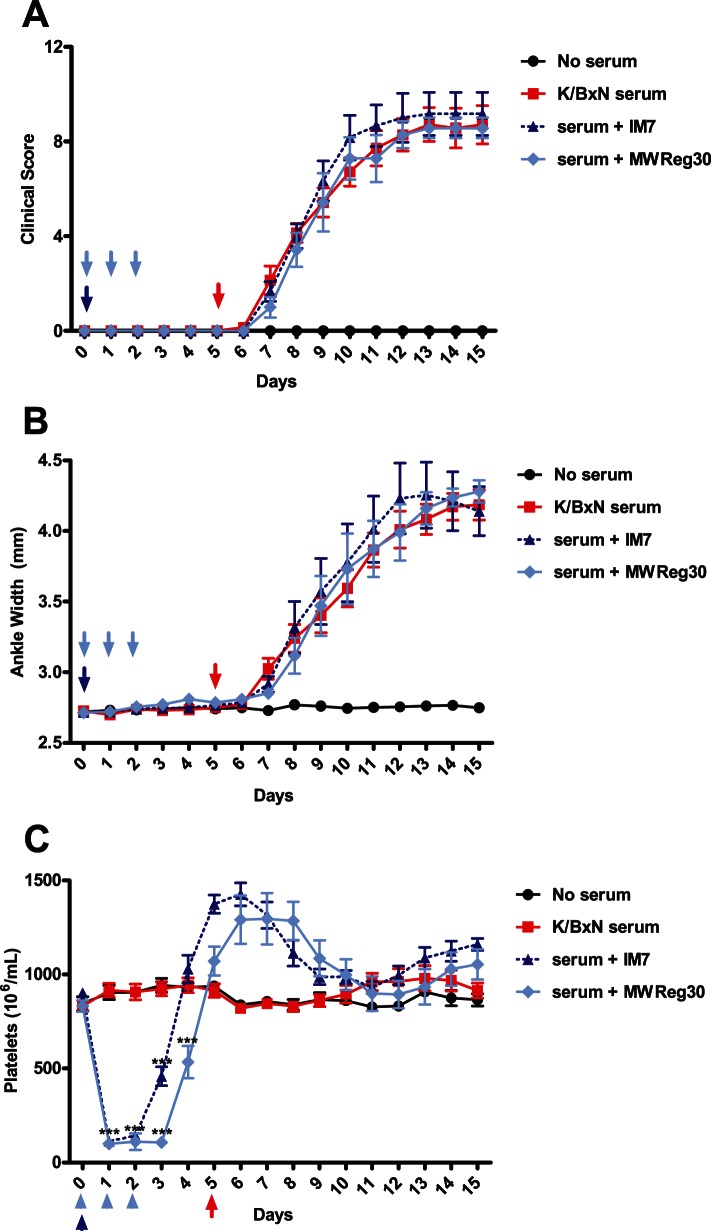
Rebound thrombocytosis is not sufficient to increase the severity or rate of development of arthritis. C57BL/6 mice were assessed for baseline measurements on day 0. Mice were then pre-treated either with a single 150ug dose of IM7 (dotted blue) on day 0 or with 3 daily doses of 2ug MWReg30 (light blue) on days 0–2. Arthritis was induced by injecting mice with K/BxN serum on day 5 after daily measurements had been taken. Clinical scoring and platelet counts were taken every 24 hours for 15 days. Neither treatment significantly influenced the progression or severity of arthritis, as measured by Clinical Scoring (A) and ankle width measurements (B). C) Both IM7 and MWReg30 induced a similar degree of thrombocytopenia. (n = 6 mice from 3 separate experiments). *P<.05, **P<0.01, ***P<0.001 by two-way ANOVA.

**Figure 6:**
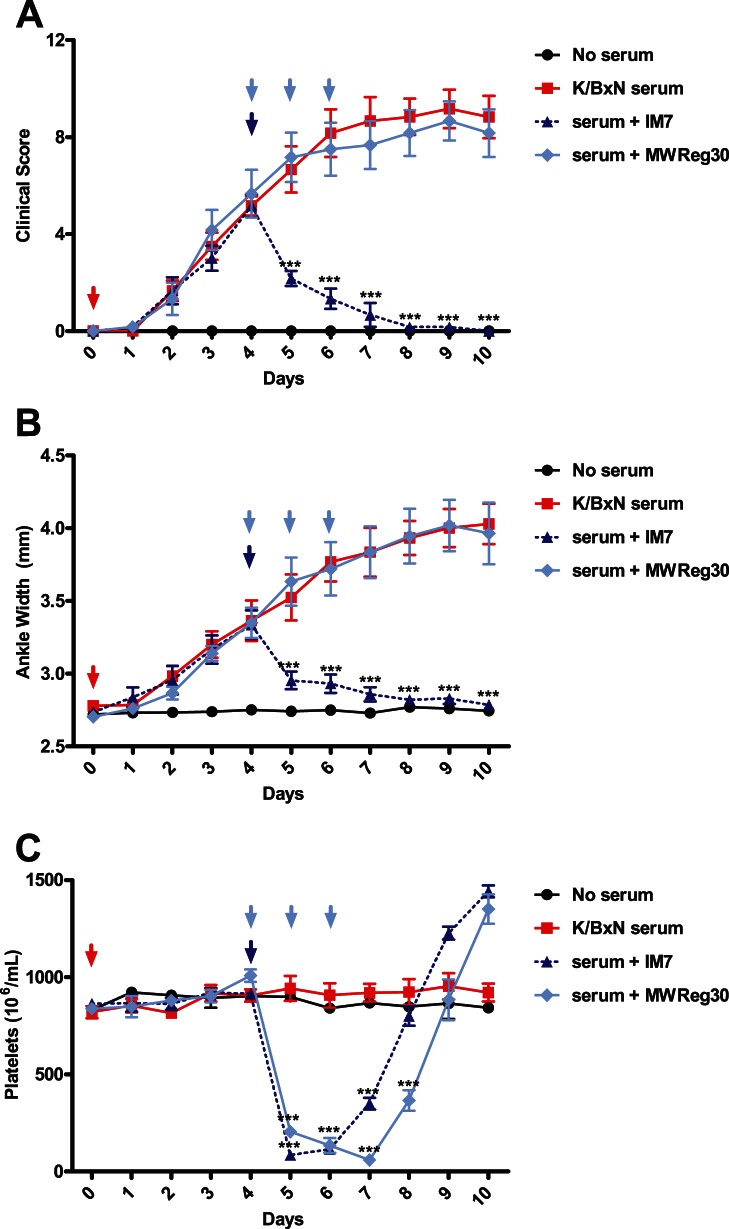
Anti-CD44, but not anti-platelet treatment, is capable of reversing the ongoing development of arthritis. C57BL/6 mice were assessed for baseline measurements on day 0, then arthritis was induced by injecting mice with 160uL of K/BxN serum. Arthritis was allowed to develop for 4 days, then mice were treated with either a single 150ug dose of IM7 (dotted blue) or 3 daily doses of 2ug MWReg30 (light blue) on days 4–6. Clinical scoring and platelet counts were taken every 24 hours for 10 days. MWReg30 had no significant effect on the progression of arthritis, while IM7 quickly reversed the developing arthritis, as measured by Clinical Scoring (A) and ankle width measurements (B). C) Both IM7 and MWReg30 induced a similar degree of thrombocytopenia. (n = 6 mice from 3 separate experiments) *P<.05, **P<0.01, ***P<0.001 by two-way ANOVA.

Prism 5.0 (GraphPad Software, Inc. La Jolla, CA) was used for the representation of data (mean ± SEM), including all statistical calculations. Statistical analyses for Figures 1 and 2 were conducted using a two-tailed, unpaired Student’s t-test. Statistical analyses for Figures 3–6 were conducted using two-way ANOVA, followed by Bonferroni correction. *P* values smaller than 0.05 were considered significant.

## Results

### Platelet Depletion Partially Protects against Serum-Induced Arthritis

Platelet-depletion has been reported to ameliorate arthritis in the K/BxN model [25], therefore we conducted a similar experiment using a monoclonal anti-platelet antibody (MWReg30, anti-GPIIb) to induce thrombocytopenia. K/BxN serum-injected mice receiving no therapeutic treatment developed joint swelling and inflammation, reaching a plateau by day seven with a clinical score of approximately 8–9, whereas K/BxN serum-injected mice treated with anti-platelet antibody developed an attenuated arthritis significantly less than arthritic controls (Figure 1A,B). While anti-platelet treatment reduced the level of inflammation (clinical score ∼5; day 10), this group still developed significant inflammation. Anti-platelet treatment from days 0–3 induced significant thrombocytopenia over the course of the treatment (Figure 1C), followed by an elevated platelet count from days 6–9, with an eventual return to baseline on day 10. Our results support the work of Boilard *et al.* [25], who noted that a polyclonal anti-platelet treatment (anti-GPIbα) reduced inflammation up to 85% relative to arthritic controls, while causing thrombocytopenia up to 6 days from induction [32].

To determine if prolonged thrombocytopenia would affect the onset and/or severity of disease, mice were injected with anti-platelet antibody continuously for 10 days. The group receiving daily injections of anti-platelet antibody experienced sustained thrombocytopenia throughout the experiment (Figure 2C). However, this treatment regimen did not further reduce the onset or severity of arthritis, as compared to mice receiving only 3 injections of anti-platelet antibody (Figures 1 and 2).

### Cd44 Antibodies Induce Thrombocytopenia and Ameliorate Arthritis

We previously determined that several CD44 antibodies could protect against passively-induced immune thrombocytopenia (ITP) while other CD44 antibodies themselves caused thrombocytopenia [22]. To examine if there was a close relationship between the ability of selected CD44 antibodies to ameliorate inflammatory arthritis and induce thrombocytopenia at relevant doses, the antibodies IM7, KM201, KM114, KM81, 5035-41.1D,IRAWB14.4, and control IgG were examined at doses and regimes similar to those used in other murine arthritis models [12], [13], [14]. Treatment with control IgG did not significantly reduce either platelet counts or inflammation (Figure 3A–C). In contrast, the IM7 CD44 antibody induced severe thrombocytopenia, which resolved approximately 4 days after the last injection, followed by a transient period of increased platelet counts (Figure 3F). IM7 administered on day 0 essentially eliminated inflammation over the first 4 days of the experiment, while the second injection on day 2 further delayed the development of arthritis, and reduced the overall level of inflammation for the duration of the experiment (Figure 3D,E).

IRAWB14.4 induced a significant but less severe thrombocytopenia as compared to IM7 treatment (Figure 3I) but also protected against arthritis (Figure 3G,H). A single injection trended towards reducing the level of inflammation relative to arthritic controls, while a second injection of IRAWB14.4 resulted in a statistically significant protection. KM81 induced significant thrombocytopenia (Figure 3L) and delayed the onset of inflammation (Figure 3J,K), though this antibody was one of the least effective in ameliorating arthritis in this model.

Mice treated with either one or two injections of 5035-41.1D experienced a moderate and non-additive drop in platelet counts (Figure 4C, solid line vs. dashed line). Despite this, 2 injections of this antibody produced the most effective reduction in the clinical score observed in this study (Figure 4A,B).

Treatment with CD44 antibody KM201 induced a moderate drop in platelet counts (Figure 4F), yet also significantly protected against inflammation for an extended period (Figure 4D,E). Treatment with KM114 induced moderate thrombocytopenia (Figure 4I) and delayed the onset of inflammation (Figure 4G,H).

While each of these CD44 antibodies induced thrombocytopenia and reduced inflammation, the length and severity of the thrombocytopenia did not appear to mirror their therapeutic effect.

### Rebound Thrombocytosis does not Accentuate Arthritis

To determine if an increased platelet count could affect the severity or course of arthritis, we treated mice with anti-platelet antibody or IM7. Both groups were followed for evidence of rebound thrombocytosis, and arthritis then induced on day 5 (Figure 5). Arthritis developed normally in mice that only received K/BxN serum, reaching a plateau at approximately 7–8 days after induction (days 12–13) as expected (Figure 5A,B). The arthritis in the anti-platelet antibody and IM7 antibody pre-treated groups were almost identical to the K/BxN-treated controls, suggesting that moderately increased platelet counts (Figure 5C) did not significantly affect the onset or clinical severity of arthritis in this model. Alternatively, it may be possible that the high circulating concentration of platelets in the blood are in sufficient number and/or concentration to contribute to their proinflammatory role in this model.

### Cd44 Antibodies, but not Anti-Platelet Antibody, Reverses Ongoing Inflammatory Arthritis

Arthritis was induced in all groups on day 0, and inflammation was allowed to progress normally (Figure 6A,B). On day 4 (midway to the plateau stage of arthritis), mice were treated with either a single injection of IM7 or anti-platelet antibody. IM7 rapidly induced severe thrombocytopenia (Figure 6C) and reversed the developing arthritis, with a 50% decrease in inflammation 24 hours after IM7 administration (day 5). Mice treated with anti-platelet antibody also developed severe thrombocytopenia (Figure 6C), but this regime did not reverse the progression or clinical severity of arthritis (Figure 6A,B), in sharp contrast to IM7.

## Discussion

Recent experiments by Boilard and colleagues elegantly demonstrated a role for platelets [25], [26] and platelet microparticles [25] in the development of inflammation using the K/BxN serum transfer model of arthritis. The K/BxN serum transfer model induces arthritis independent of adaptive immunity [30], but reliant upon innate immunity [33]. In agreement with the findings of Boilard and coworkers, we also observed here that an anti-platelet antibody was effective in inhibiting the development of inflammatory arthritis. A key difference between the anti-platelet antibody used by Boilard *et al* and the anti-platelet antibody used in this study is that they used a polyclonal antibody specific for GPIb, while we used a monoclonal antibody specific for GPIIb. Different platelet membrane glycoproteins (e.g., GPIb and GPIIb) are linked to distinct platelet functional events, but interestingly, antibodies specific to either of these glycoproteins can be effective in this model. We speculate therefore that the net reduction in circulating platelets is more likely responsible for the anti-inflammatory effects, rather than a mechanism related to blocking the function of the particular platelet antigen targeted.

We have previously demonstrated that IM7 (an anti-CD44 commonly used in ameliorating murine models of inflammatory arthritis [11], [12], [13]) can induce thrombocytopenia on its own [22]. Given that immune thrombocytopenia (induced by anti-platelet treatment) can ameliorate inflammatory arthritis, we hypothesized that the ameliorative effects of anti-CD44 treatment in inflammatory arthritis could potentially be mediated by their ability to cause thrombocytopenia. However, the relative ability of each CD44 antibody to ameliorate arthritis onset, or clinical severity, did not appear to be closely linked to their ability to induce thrombocytopenia. As well, continuous depletion of platelets with daily injection of anti-platelet antibody did not increase the protection against arthritis. In contrast, an additional injection of most anti-CD44 antibodies increased the protection against arthritis. In particular, a single IM7 treatment eliminated visible inflammation for five days, and an additional dose of this antibody prolonged protection against arthritis. These findings do not support the concept that CD44 antibodies have anti-inflammatory effects primarily mediated by a thrombocytopenic mechanism.

For amelioration of ongoing arthritis, immune depletion of platelets was not able to reverse ongoing inflammation once arthritis had already been induced; in contrast, IM7 quickly and effectively reversed ongoing arthritic inflammation. This may indicate that platelets are more likely involved in disease initiation, while the anti-inflammatory pathway initiated by IM7 can reverse established disease. IM7 has been shown to be effective in several different animal models of autoimmunity [12], [14], [34], [35]. This antibody can induce the formation of platelet-neutrophil complexes in proteoglycan-induced arthritis (PGIA) [14], but whether this is related to the effects seen in this study are unknown.

Unlike IM7, IRAWB14.4 has been shown to significantly enhance CD44 binding to HA, possibly through cross-linking of adjacent CD44 molecules [36]. Previous work has shown it to be ineffective in treating arthritis in the PGIA model [13], possibly due to its ability to increase the binding of lymphocytes [36] and neutrophils [37] to HA. While these facts are in contrast to IRAWB14.4′s effectiveness observed in our model, the PGIA model relies on both adaptive and innate immunity [38], whereas the K/BxN model only involves innate immunity [33]. While speculative, it is possible that anti-CD44 treatment in PGIA may have a markedly different mechanism than anti-CD44 treatment in K/BxN.

Of all the antibodies examined in this study, 5035-41.1D was arguably the most effective in inhibiting inflammation, significantly reducing both the rate of inflammatory development and severity of arthritis. A single dose of 5035-41.1D induced a very mild thrombocytopenia, and two doses did not significantly increase the length of the thrombocytopenic period, in contrast to the additive effects of both IM7 and IRWAB14.4. There is no published data regarding the ability of 5035-41.1D to affect binding of CD44 to HA that we are aware of. The fact that two doses of 5035-41.1D increased the protection against arthritis without further decreasing the platelet count supports our interpretation that anti-CD44 treatment does not likely act solely through inducing thrombocytopenia. In fact, 5035-41.1D at lower doses can actually prevent immune thrombocytopenia in a mouse model of ITP [22]. We also note that this is the first description of the ability of 5035-41.1D to ameliorate arthritis in any model of inflammatory arthritis.

The KM-group of antibodies used in this study are thought to have an overlapping function, unlike some of the other anti-CD44 antibodies we examined here. Instead of primarily modulating the surface expression of CD44, or aggregating CD44 molecules, they bind to epitopes on CD44 in the hyaluronan binding pocket [21], and are thought to interrupt platelet [39] and leukocyte [18], [40], [41] binding to HA. KM201 has been used to successfully treat the PGIA-model of arthritis [13], though our results indicated that this antibody is significantly more effective in the K/BxN model, even at 50% of the dosage used in the PGIA model. KM114 was similarly effective in ameliorating arthritis, and while two doses increased the period of amelioration, the second dose did not significantly affect the thrombocytopenia. Similar to IM7, KM81 has been shown to reduce the number of granulocytes circulating in the periphery [14], providing further support for the concept that CD44 antibodies may be treating arthritis by a mechanism that does not rely solely on platelet depletion.

Unlike mice, human platelets do not appear to express CD44 [42] thus development of a CD44 antibody to treat RA would not be expected to induce thrombocytopenia in humans. A full understanding of the mechanism of anti-CD44’s therapeutic effect(s) will be critical to the development of an effective treatment. While we are unable to rule out the possibility that antibody-induced thrombocytopenia contributes to some of the therapeutic effect, it seems reasonable to suggest that thrombocytopenia-independent mechanisms likely contribute to the primary ameliorative actions of CD44 antibodies.

To our knowledge this is the first study which has examined IM7, KM201, KM114, KM81, 5035-41.1D, and IRAWB14.4 in the same model, and we have shown that all of these CD44 antibodies delayed the onset and reduced the clinical severity of arthritis in this model. This is an interesting finding given that these antibodies represent different IgG isotypes; binding epitopes [21], can interfere [13], [18], [39], [40], [41] or augment [36] HA binding to CD44 on some cells, and/or cause proteolytic cleavage and shedding of the CD44 molecule [13], [43]. It is possible that the CD44 antibodies used here work by more than one discrete mechanism in arthritis, with different antibodies falling into different classes of effector molecules or that the antibodies may of course all work by different unrelated mechanisms. Notwithstanding these potential differences between the various antibodies employed in the study, it seems unlikely that the thrombocytopenic effect of these antibodies contribute substantially to their ameliorative effects in inflammatory arthritis.
